# Translational Biomedical Informatics and Computational Systems Medicine

**DOI:** 10.1155/2013/237465

**Published:** 2013-12-02

**Authors:** Zhongming Zhao, Bairong Shen, Xinghua Lu, Wanwipa Vongsangnak

**Affiliations:** ^1^Department of Biomedical Informatics, Vanderbilt University School of Medicine, Nashville, TN 37203, USA; ^2^Center for Quantitative Sciences, Vanderbilt University Medical Center, Nashville, TN 37232, USA; ^3^Department of Psychiatry, Vanderbilt University School of Medicine, Nashville, TN 37212, USA; ^4^Department of Cancer Biology, Vanderbilt University School of Medicine, Nashville, TN 37232, USA; ^5^Center for Systems Biology, Soochow University, Suzhou, Jiangsu 215006, China; ^6^Department of Biomedical Informatics, University of Pittsburgh, Pittsburgh, PA 15206, USA

Translational biomedical informatics is rapidly emerging as a new discipline to meet translational medical research demands. This discipline integrates a variety of data from medical research, biological research, and electronic medical records. Computational systems medicine applies computational and systems biology approaches to solve complex problems in medical research; this approach aims for a deeper understanding of disease pathophysiology and a systems level view of disease development. Systems medicine approaches assist investigators with better biomarker discovery and, thus, improve the diagnosis, prognosis, and treatment of complex diseases. Research activities in these areas have rapidly expanded, largely due to the huge volume of data generated from high throughput technologies such as next-generation sequencing (NGS), availability and better management of the massive amount of clinical data, and the demand to effectively link biological and genetic data to clinical records. One example is the B2B program, which includes two iterative components: bench-to-bedside, such that the basic research findings can be translated to clinical practice, and bedside-to-bench, such that the refinements to clinical practice offer new clinical insights and samples for experimental investigation. These complimentary components further enhance translational applications. Among the activities of translational biomedical research and clinical practice, computational approaches, including data curation and management, algorithm and model development, multidimensional data integration, data visualization, and high performance computing, provide fundamental support.

We launched this special issue to address the demand for translational biomedical informatics and discuss the current advances in this field. We are interested in both new theories and tools in this area as well as their applications in translational research. We specifically encouraged the submission of work in areas such as “-omics” data integration and analysis in complex diseases, NGS data analysis and application in medicine, systems biology related research in biomedicine, biomedical data mining and database management, system level modeling and simulation of complex diseases, and visualization of complex data in medicine. Correspondingly, after a rigorous peer review, six papers were selected from the 12 submissions. We briefly describe these papers as follows.

In “*Translational bioinformatics for diagnostic and prognostic prediction of prostate cancer in the next-generation sequencing era,*” J. Chen et al. discussed the current technological advances in molecular biomarker discovery and their translation into the clinical realm for prostate cancer diagnosis and prognosis. The authors reviewed the advances and challenges in the discovery of molecular markers for diagnosis and prognosis of prostate cancer based on high throughput technologies, including microarray and NGS. The authors highlighted 24 prostate cancer NGS studies and discussed prostate cancer biomarkers at the pathway level. Finally, they provided future direction and perspectives on translational research in prostate cancer.

In “*Exploring the cooccurrence patterns of multiple sets of genomic intervals,*” H. Wu and Z. S. Qin presented a novel statistical method and software tool to characterize the cooccurrence patterns of multiple sets of genomic intervals found in high throughput data such as ChIP-seq. Specifically, they applied a finite mixture model to measure co-occurrence patterns and demonstrated the model's accuracy using simulation and real data. The method is useful to detect co-occurrence patterns in genomic interval-based large datasets.

In “*Diagnosis value of the serum amyloid A test in neonatal sepsis: a meta-analysis,*” H. Yuan et al. performed a meta-analysis of the serum amyloid A (SAA) test as a diagnostic marker for neonatal sepsis. Neonatal sepsis is a common human disorder. It is caused by a bacterial blood stream infection in a newborn baby, which produces a high fever. Through a meta-analysis of studies retrieved from PubMed, EMBASE, the Cochrane Library, and the Google Network between January 1996 and June 2013, the authors found a moderate accuracy of a SAA test in the diagnosis of neonatal sepsis, suggesting that SAA might be promising for the diagnosis of neonatal sepsis.

In “*Characterization of schizophrenia adverse drug interactions through a network approach and drug classification,*” J. Sun et al. first constructed a schizophrenia-specific adverse drug interaction network and then characterized the schizophrenia and adverse drug interactions using the Anatomical Therapeutic Chemical (ATC) classification system. The authors found that schizophrenia drugs tend to have more adverse drug interactions than other drugs. They further revealed the distinct biological features of schizophrenia typical and atypical drugs. This work is the first to characterize the adverse drug interactions in the course of schizophrenia treatment.

In “*CADe system integrated within the electronic health record,*” N. Vállez et al. implemented an electronic health record (EHR) combined with a computer aided detection (CADe) system for breast cancer diagnosis, aiming to provide radiologists with a comprehensive working environment that facilitates the integration, image visualization, and use of aided tools within the EHR. The CADe system allows a user to display, edit, and report results in standardized formats not only for the patient information but also for their medical images. More features will be added in future work.

In “*NCBI2RDF: enabling full RDF-based access to NCBI databases,*” A. Anguita et al. introduced the NCBI2RDF system that provides users with RDF-based access to the complete NCBI data repository. RDF is a standard model for data interchange on the Web and was created by the W3C consortium and accepted as a standard in 2004. The NCBI2RDF, which has two steps (metadata generation and query resolution), enables a user to obtain integrated access to comprehensive data within other existing RDF-based repositories, overcoming current limitations on NCBI data search by implementing its Entrez system.

